# Discovering Immune-Mediated Mechanisms of Gastric Carcinogenesis Through Single-Cell RNA Sequencing

**DOI:** 10.3389/fimmu.2022.902017

**Published:** 2022-06-10

**Authors:** Stella G. Hoft, Michelle D. Pherson, Richard J. DiPaolo

**Affiliations:** ^1^ Department of Molecular Microbiology and Immunology, Saint Louis University School of Medicine, St. Louis, MO, United States; ^2^ Department of Biochemistry and Molecular Biology, Saint Louis University School of Medicine, St. Louis, MO, United States; ^3^ Genomics Core Facility, Saint Louis University School of Medicine, St. Louis, MO, United States

**Keywords:** gastric cancer, single cell RNA sequencing (scRNAseq), T cell, B cell, myeloid cell, type 2 innate lymphoid cell (ILC2), mast cell, stromal cell

## Abstract

Single-cell RNA sequencing (scRNAseq) technology is still relatively new in the field of gastric cancer immunology but gaining significant traction. This technology now provides unprecedented insights into the intratumoral and intertumoral heterogeneities at the immunological, cellular, and molecular levels. Within the last few years, a volume of publications reported the usefulness of scRNAseq technology in identifying thus far elusive immunological mechanisms that may promote and impede gastric cancer development. These studies analyzed datasets generated from primary human gastric cancer tissues, metastatic ascites fluid from gastric cancer patients, and laboratory-generated data from *in vitro* and *in vivo* models of gastric diseases. In this review, we overview the exciting findings from scRNAseq datasets that uncovered the role of critical immune cells, including T cells, B cells, myeloid cells, mast cells, ILC2s, and other inflammatory stromal cells, like fibroblasts and endothelial cells. In addition, we also provide a synopsis of the initial scRNAseq findings on the interesting epithelial cell responses to inflammation. In summary, these new studies have implicated roles for T and B cells and subsets like NKT cells in tumor development and progression. The current studies identified diverse subsets of macrophages and mast cells in the tumor microenvironment, however, additional studies to determine their roles in promoting cancer growth are needed. Some groups specifically focus on the less prevalent ILC2 cell type that may contribute to early cancer development. ScRNAseq analysis also reveals that stromal cells, e.g., fibroblasts and endothelial cells, regulate inflammation and promote metastasis, making them key targets for future investigations. While evaluating the outcomes, we also highlight the gaps in the current findings and provide an assessment of what this technology holds for gastric cancer research in the coming years. With scRNAseq technology expanding rapidly, we stress the need for periodic review of the findings and assess the available scRNAseq analytical tools to guide future work on immunological mechanisms of gastric carcinogenesis.

## Introduction

Gastric cancer remains one of the leading causes of cancer-related mortality worldwide (1). Gastric adenocarcinomas make up the majority of all gastric cancers. Adenocarcinomas are usually classified using the clinically/histologically defined Lauren classification system or the molecularly defined The Cancer Genome Atlas (TCGA) and Asian Cancer Research Group (ACRG) subtypes (2–4). The Lauren classification system identifies two subtypes of gastric adenocarcinoma, *viz.*, intestinal and diffuse; the latter has very poor clinical outcomes (2). TCGA, with similar results from the ACRG, defines four subtypes of adenocarcinoma, *viz.*, 1. EBV-positive, 2. microsatellite instability, 3. genomically stable, and 4. chromosomal instability (3, 4). While these new classifications are clinically useful for distinguishing different subtypes of gastric cancer for different treatment modalities, additional studies are needed to understand how gastric adenocarcinomas of all subtypes arise, which is crucial in designing effective strategies for targeted preventative medicine. Chronic gastric inflammation, caused by *Helicobacter pylori* infection, is the most common instigator of gastric epithelial cell transformation into precancerous lesions and eventually into dysplastic tumors (5, 6). We know well that the prominent immune response in the early stages of chronic gastritis is made up of IFN-γ-producing CD4^+^ T-helper 1 cells (Th1s) and IL-17-producing CD4^+^ T-helper cells (Th17s.) (7–11) While conventional studies have established that these T cells and the cytokines they secrete induce gastritis and tissue damage, scRNAseq technology now allows for a much broader and unbiased investigation of the immune landscape, including immune cells, cytokines, and inflammatory triggers that regulate the onset of gastric cancer. This thereby helps to focus our attention on specific mechanisms that impact tumorigenesis.

The advent of scRNAseq technology has revolutionized examining cellular mechanisms at single-cell resolution and mechanisms at the molecular level across many disciplines.

The exceptional success of this technology prompted many reviewers to provide helpful overviews of its usefulness in investigating cancers and GI diseases, as this review now focuses on the immune-mediated mechanisms of gastric carcinogenesis (12–18). While the first scRNAseq was performed by Tang et al. as recently as 2009, the widespread use of scRNAseq technology in the last several years has inspired developing many convenient protocols with specific tasks, which are rapidly made commercially available to researchers (19). This easy access has contributed to an explosive increase in scRNAseq publications as well as a rapid expansion of refined analytical capabilities. These trends can be clearly seen within the field of gastric cancer, with almost forty publications in the last few years. Among the published scRNAseq gastric cancer works, it is apparent that they have undertaken various approaches to their studies. Most groups obtained primary gastric cancer tissues from patients to generate scRNAseq data (20–35). Some utilized previously published primary tumor datasets for further analysis (36–43). Others transcriptionally profiled “liquid biopsies” of metastatic ascites fluid taken from gastric cancer patients (44, 45). Realizing that human tissues are difficult to procure, some created laboratory models of gastric diseases, either as *in vivo* mouse settings or *in vitro* gastric organoids (35, 46–55). Beyond the purview of this review, other valuable scRNAseq datasets also have been generated from healthy gastric tissues (56–59).

**Figure 1:**
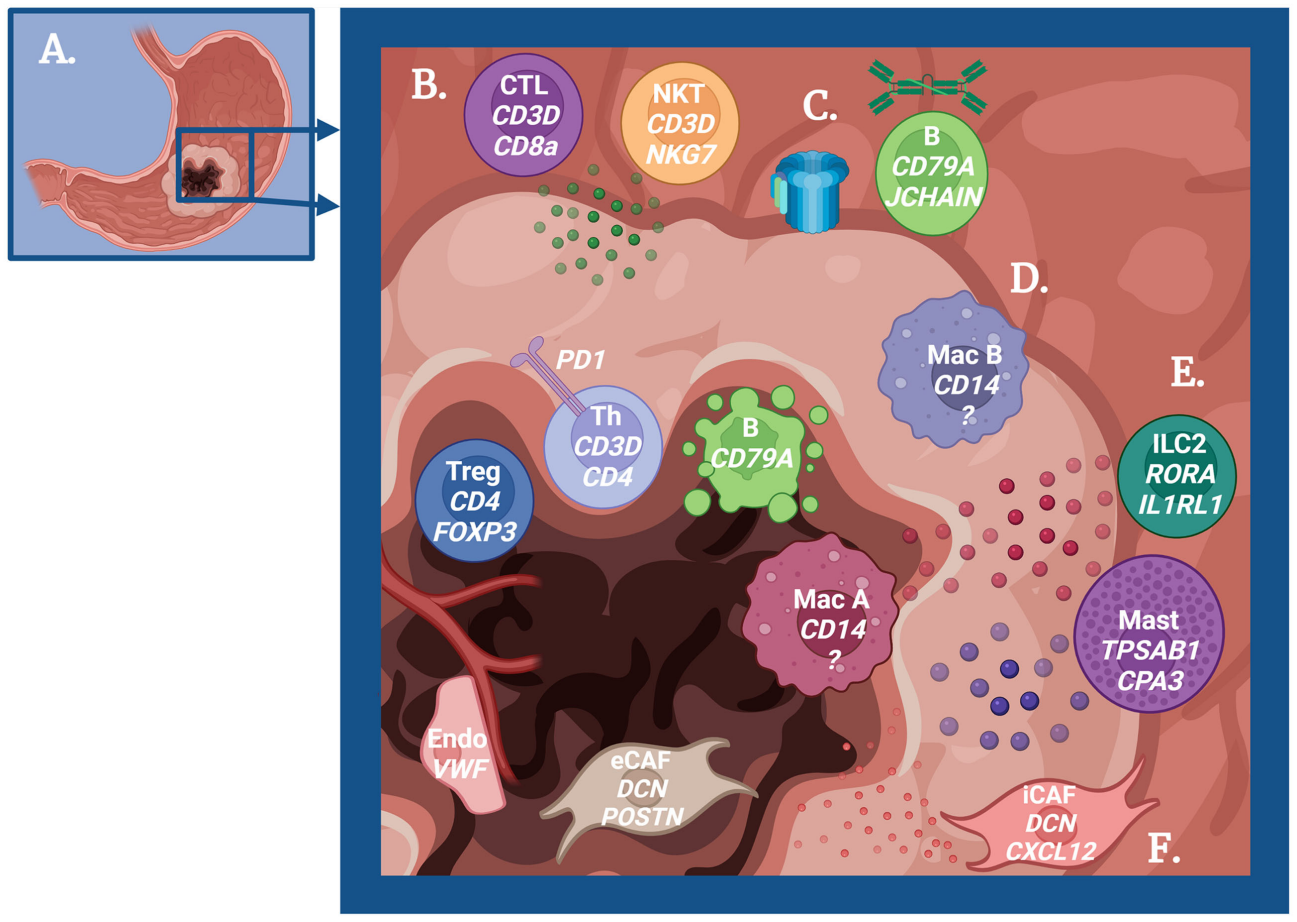
Schematic overview of the immune-mediated mechanisms in gastric carcinogenesis. **(A)** Cartoon depicting a stomach with gastric adenocarcinoma. **(B)** Role of T cells in tumor immunity. Peripheral Cytotoxic T cells (CTL) and Natural Killer T cells (NKT) produce granzyme to promote antitumor immunity, but regulatory T cells (Treg) and exhausted T helper cells (Th) are dominant near the tumor center. **(C)** Role of B cells in tumor immunity. B cells promote antitumor immunity *via* IgA production and complement activation early in the pathogenesis, but these B cells undergo apoptosis as cancer progresses. **(D)** Role of macrophages in tumor immunity. Some macrophages (M1-like) promote antitumor immunity, while other macrophages (M2-like) promote tumor growth. **(E)** Role of ILC2s and mast cells in tumor immunity. The cytokine IL-13 produced by ILC2s and mast cells may promote tumor progression and act on tumor-promoting macrophages. Mast cells also produce histamine, which has a potential role in cancer progression. **(F)** Role of fibroblasts and endothelial cells in tumor immunity. Inflammatory cancer-associated fibroblasts (iCAF) regulate immune cells to support tumor growth, while epithelial to mesenchymal CAFs (eCAF) directly promote tumor growth. Endothelial cells similarly play a role in immune regulation to increase metastatic potential. Created with Biorender.com.

As the scRNAseq publications in gastric diseases continue to grow, we take a comprehensive look at the published work to assess how far we have come and how far we can go. As summarized in **Figure 1**, across all scRNAseq gastric disease datasets, the identified subsets of cells that play a role in the tumor microenvironment (TME) include T cells, B cells, macrophages, mast cells, ILC2s (innate lymphoid cells-type 2), fibroblasts, and endothelial cells. Herein, we discuss how they were identified using scRNAseq and their immunological roles in driving/impeding cancer progression. Furthermore, we assess the newly discovered response of epithelial cells, either malignant or non-malignant, to inflammation. Finally, we overview the available scRNAseq analytical tools and evaluate how they can be better utilized in gastric disease research.

## T Cells

T cells are the most identified immune cells across all gastric cancer scRNAseq datasets using *CD2* and *CD3D*. ScRNAseq analysis by Li et al. (2022) reveals T cells as the most highly enriched immune cell population in the tumor microenvironment (TME), and their ratio increases as the disease advances (24). A greater diversity of unique T cell subsets and T cell receptor (TCR) repertoire were identified within tumor tissues when compared with that of patient-matched healthy tissue or peripheral blood mononuclear cells (PBMCs), suggesting that a variety of unique T cells infiltrate the TME (22). The main subsets of T cells that show infiltration into the TME in gastric cancer scRNAseq datasets include CD4^+^ T helper cells (Th, co-expressing *CD4*), CD8^+^ cytotoxic T lymphocytes (CTL, co-expressing *CD8A*), regulatory T cells (Treg, co-expressing *FOXP3*), and natural killer T cells (NKT, co-expressing *NKG7*) (20, 22, 26, 39). Among these infiltrated subsets, some T cells have been implicated in a tumor-promoting role and others in a tumor-inhibiting role. CD4^+^ central memory precursor T cells (expressing *CCR7, SELL, IL7R*) were the most abundant subtype in tumor tissue, whereas the effector and effector memory T cells were abundant in the adjacent tissue (24). Similarly, a large number of Tregs showed infiltration into the tumor tissue compared to the adjacent tissue. Along with all these findings, Jiang et al. (2022) and Kumar et al. (2022) confirmed that with tumor progression the tissue microenvironment changes from predominance of pro-inflammatory effectors to predominance of immunosuppressive Tregs (34, 35). Qu et al. (2022) identified a distinct Treg phenotype that is increased in tumor tissue compared to patient-matched blood and defined by enriched expression of the TNF receptor superfamily member 1B (*TNFR2*) (30). *TNFR2* ^+^ Tregs were found to be functionally immunosuppressive and an increased infiltration into tumor tissue correlated with poor prognosis. An interesting study by Jeong et al. (2021) investigated the nature of T cells along the depth of tumor tissue, from the surface layers to deep layers of diffuse gastric tumors, and found that the greater the depth of T cell penetration the greater the change in their functional phenotype–from naïve to exhausted T cells (21). This remarkable relationship of tumor depth vs. T cell exhaustion also correlates with a gradient of *CCL2* within the tumor tissue. *CCL2* production by fibroblasts and endothelial cells in the deep TME correlates with T cell exhaustion. Furthermore, the deepest layer of tumor tissue is enriched in Tregs, indicating that the deep TME promotes T cell exhaustion as well as immunosuppression, perhaps supporting tumor protection. These scRNAseq studies indicate that the TME in gastric cancers abundantly recruits T cells and at the same time, remodels their functional phenotype, tilting the balance toward an immunosuppressive pro-tumor milieu.

Autophagy, a self-degradative process, has been associated with tumor progression (60). Tong et al. (2021) observed an enhanced autophagy signature in scRNAseq data that increased with gastric disease progression, which could be used to predict patients at high risk of succumbing to the disease (38). On the other hand, a low-risk autophagy score of gastric cancer correlated with an increase in the levels of activated Ths and CTLs, expression of PD-1/CTLA-4 transcripts, and a decrease in the prevalence of Tregs. Collectively, these findings suggest that an intensified autophagy pathway within gastric tumors hinders T cell effector function. With decreased activation of effectors, T cells are less likely to become exhausted, which, in turn, limits the efficacy of immunotherapy, further promoting immune tolerance. Therefore, this autophagy pathway could be another effective target for future gastric cancer therapies in combination with immunotherapy.

The exhausted CD4^+^ Ths in the TME, understandably, have poor expansion function. In this situation, other T cell subsets, like NKTs and CTLs, appear to have increased TCR clonotype amplification, suggesting that they might play essential roles in the antitumor T cell response (22). Studies show that NKT cells are abundant in gastric cancer tissue with matured tertiary lymphoid structures, whereas CTLs are abundant in tumor tissue lacking lymphoid structures, suggesting that CTLs may be the earlier effectors, but NKTs may have a greater role later in orchestrating antitumor immune cells. A study that examined the CTLs within a rare type of gastric cancer, hepatoid adenocarcinoma of the stomach, found the CTLs with an exhausted phenotype; therefore, NKT cells may be more capable of long-standing cytotoxic effector function (25). There was also an enrichment in exhausted CTLs in patients with metastatic compared to non-metastatic gastric cancer correlating with worse survival outcomes (34). Fu et al. (2020) found there was decreased expression of the transcription factor IRF8 in cancer CTLs compared to normal tissue CTLs, thereby hinting that this method could be a new way to identify exhausted CTLs (20). Soon after, it was discovered that a low expression of IRF8 in gastric cancer patient PBMCs correlated with enhanced disease progression. Taken together, these observations indicate that advancing gastric cancer microenvironments reduce IRF8 expression in CTLs and suppress antitumor immunity by promoting exhaustion. In patients treated with chemotherapy, there was an observed increase in expression of *LAG3*, an immune checkpoint molecule, within the tumor tissue of non-responders, suggesting chemotherapy may contribute to CTL exhaustion (33). Among responders to chemotherapy, however, there was an observed increase in NK cell, or possibly NKT cell, tumor infiltration. These findings point toward the need for increased research into NKT cells as cytotoxic effectors, possibly being more resilient to exhaustion than CTLs. Kwon et al. (2021) similarly found that patients who responded to immunotherapy had expanded CTLs and NK populations compared to non-responders (32). When further classifying the post-treatment CTLs, there was an observed increase in exhausted CTLs, but in responders these showed high proliferation phenotypes whereas in non-responders, exhausted CTLs appeared terminally differentiated. This finding suggests that in some patients exhausted CTLs have the capability to proliferate and reactivate after successful immunotherapy, but further work defining the role of NK and NKT cells in antitumor immunity is needed to improve mechanistic understanding and treatment options for gastric cancer.

Various mouse models have been employed for scRNAseq analyses to gain insights into the specific role of Th cells in initiating disease and promoting epithelial cell transformation into precancerous and dysplastic cells. In a mouse model of autoimmune gastritis, Bockerstett et al. (2020) conducted scRNAseq in precancerous gastric lesions and found that IL-27, likely produced by macrophages and dendritic cells, acts on Th cells to suppress inflammation and limit disease progression (48). It appears that IL-27 is important for preventing carcinogenesis in the early stages of the disease. However, in models where mice develop similar precancerous lesions, either induced by deprivation of androgen and glucocorticoids or acute drug injury, T cells were not required for disease progression (50, 61). This suggests that while T cells and the cytokines they produce induce tissue damage, additional cell types might be needed to drive epithelial cell injury and transformation (62, 63). Similarly, as human gastric cancer is typically brought about by chronic inflammation, it is likely that T cells initiate gastritis with early tissue damage and other cell types take over promoting cancer development and progression (5, 6). Nagaoka et al. (2020) generated a gastric cancer cell line tumor model in mice, performed scRNAseq analysis, and found that successful tumor growth required *Il17a* in tumor-infiltrating CD4+ Ths (54). Consequently, treating the tumor-inoculated mice with a combination of anti-IL-17a and anti-PD-1 eliminated 80% of the tumors. This suggests that combinational therapeutic approaches that also target T cells could benefit the patients who are unresponsive to immune-checkpoint blockade alone. Considering all the new findings, scRNAseq technology has been incisive; it has provided new insights that Ths are important drivers of tissue damage and epithelial cell malignant transformation early in the disease, a convenient window for targeting immunotherapy and arresting the disease early.

## B Cells

B cells have been identified using *CD79A* or *MS4A1* as a prevalent subset of immune cells in scRNAseq gastric cancer datasets. In a mouse model of autoimmune gastritis, which progresses to develop precancerous and dysplastic lesions, B cells are the predominant immune cells present in the gastric mucosa (9, 48, 64). This suggests that B cells may be promoting early gastric tissue injury driving cancer progression in the setting of autoimmunity. Other models of precancerous lesions in mice, either by inducing *via* adrenalectomy or acute drug injury, found that B cells were not required for disease progression. These observations suggest that the B cells may act in parallel to promote tumorigenesis, but their role might be dispensable (50, 61). Once cancer is established in the tissue, the proportion of B cells decreases with disease progression (24). Additionally, the B cell phenotype changes from a proinflammatory state in the early stages of disease toward a pro-apoptotic state in advanced gastric cancer. Another study found that naïve B cells within tumor tissue even upregulate apoptosis-associated transcripts significantly more than the naïve B cells in the adjacent tissue (39). The B cell activating receptor, CD40, was downregulated in B cells isolated from gastric cancer patients compared to healthy individuals (20). When looking at various layers of the diffuse gastric tumors, B cells were depleted as the tumor depth increased (22). Interestingly, Kumar et al. (2022) found a significant enrichment of plasma B cells in diffuse-type tumors over intestinal tumors due to increased tumor expression of the plasma cell recruiting molecule, *KLF2* (35). While there may be more B cells recruited to diffuse tumors, there is still a consistent decrease in plasma cells as tumor grade and stage increase. Kim et al. (2022) also found that after chemotherapeutic treatment, non-responders have an observed increase in proportion of tumor infiltrating B cells, suggesting a tumor-associated cell type, not tumor cells directly, are responsible for triggering B cell apoptosis (33). Altogether, these data indicate that B cells are prevalent and capable of contributing to malignant transformation in early gastritis, but as the tumor develops, the TME promotes B cell apoptosis, thus limiting their contributions to tumor immunity.

To investigate the specific role that B cells play in tumor growth, Jia et al. (2021) focused on the subset of mucosa-associated lymphoid tissue (MALT)-derived B cells (22). MALT B cells, identified by significant upregulation of immunoglobulin-related transcripts (e.g., *JCHAIN, IGHG1*), were prevalent in tumor tissue but absent in patient-matched PBMCs. As determined by gene expression, a predominant function of these MALT B cells was related to complement activation. High expression levels of *IGHA*, *JCHAIN*, and complement activation receptors suggest that MALT B cells may be promoting antitumor immunity through activating the complement pathway. IgA, although not classically thought of as such, has been identified as a complement-activating immunoglobulin (65). As an alternative to B cells directly activating complement, a recent scRNAseq study in breast cancer found complement signaling sensed by B cells to be associated with B cell-mediated activation of effector T cells (66). Sathe et al. (2020) found that as gastric tissue transforms from healthy to tumor, B cells undergo isotype switching, changing from a previously IgA-dominant environment into an IgG-dominant environment (26). This shift from IgA to IgG could impact B cell antitumor immunity by limiting IgA-mediated complement activation in late-stage disease. B cells in gastric cancer were also found to orchestrate immune function by producing *IFNG*, *CCL3*, and *IL-8* (20). This is clear evidence that B cells may be necessary for promoting migration of antitumor immune cells. These findings suggest that the main role of B cells in limiting tumor progression lies in coordinating complement-mediated lysis and/or immune cell recruitment into the TME.

When comparing gastric cancer subtypes by survival outcome, a greater prevalence of B cells is observed in more prognostically favorable tumors. Wang et al. (2021) conducted a thorough and sophisticated analysis of peritoneal fluid from metastatic gastric cancer patients and identified unique subsets of gastric cancer based on inferred tumor cell lineage (44). The most common subsets identified were gastric-lineage dominant and GI-mixed-lineage dominant. In this study, GI-mixed-lineage tumors provided a significantly better long-term survival than gastric-lineage tumors. GI-mixed tumors were estimated to have a significantly higher abundance of B cells over gastric-dominant tumors, suggesting that either B cells are important for limiting gastric cancer growth or B cell apoptosis does not occur in less severe GI-mixed-lineage tumor environments. Looking at EBV^+^ gastric tumors as opposed to EBV^-^ tumors, B cells were shown to have higher levels of cell cycle signatures (28). This is likely because EBV infection of B cells promotes cell cycling and inhibits apoptotic pathways. This may explain why EBV^+^ tumors typically have a more favorable prognosis over EBV^-^ tumors, as B cells are less likely to undergo apoptosis and can continue promoting antitumor immunity (67). The increased prevalence of B cells in lower-risk gastric tumors suggests that novel clinical strategies could be designed to prevent B cell apoptosis in cancer patients. In future scRNAseq work, a focus on B cell subtypes present in different severities of gastric disease will be needed. Looking at how B cells from early gastritis, possibly driving tissue transformation, are phenotypically distinct from those present in early cancer, that may be important for orchestrating the antitumor immune response. As well, scRNAseq could be utilized to identify uniquely enriched transcripts in the advanced TME that are promoting B cell apoptosis, as a potential novel target for therapeutics.

## Myeloid Cells

Typically, tumor-associated macrophages (TAMs), identified in scRNAseq datasets by *CD68* and *CD14* expression, are divided into two distinct polarization states serving opposing roles (68). M1 macrophages, also considered classically activated macrophages, are known to be proinflammatory and promote antitumor immunity. M2 macrophages, also known as alternatively activated macrophages, can be anti-inflammatory and may suppress antitumor immunity. In acute drug-injury mouse models, M2s have been postulated as the main immune cell type that promotes precancerous metaplastic lesions (61). Prior to the scRNAseq studies, the macrophage types were identified with a protein marker, the scavenger receptor CD163, which is differentially enriched in M2s over M1s (69). However, in many scRNAseq studies looking specifically at the immune cells of gastric cancer tissue, the subsets of macrophages do not distinctly cluster based on M1/M2 polarization but fall into other discrete phenotypes where the same clusters do co-express M1 and M2 markers (21, 26, 34, 39, 45, 70). These results suggest that TAMs may be more diverse than previously thought. To overcome the intricacies, some groups developed alternative methods for identifying M1s and M2s in scRNAseq datasets. Among them, Eum et al. (2020) generated signature gene lists from isolated, *in vitro* differentiated, and sequenced M1 or M2 cells and then compared with the TAMs isolated from gastric cancer patients’ metastatic ascites samples (45). Using these signature gene sets, they show that TAMs indeed more closely resemble M2s than M1s and patients with a higher overall M2 signature have worse survival outcomes. This enhanced M2 signature also appears to be specific to gastric cancer over other cancer types like breast and colorectal cancers (45). Because this study looked only in patients with metastatic disease, it may be that this enhanced M2 phenotype is more prominent in metastatic tumors. Wang et al. (2021) used similar M1/M2 reference data to identify M1s and M2s in scRNAseq data and arrived at a conclusion that the fraction of M1-like macrophages is higher in a more favorable gastric cancer classification. In contrast, the M2-like macrophage fraction dominates in a less favorable classification (44). Kim et al. (2022) utilized previously published M1/M2 signature datasets to conclude that after chemotherapy there is an increase in M1-like TAMs and a decrease in M2-like TAMs, suggesting chemotherapy improves the anti-tumor TAM phenotype in patients who respond to treatment (33). Yet Li et al. (2022) used the increased expression of *CD163* to define one TAM cluster as M2s and found that the gene signature of these cells was associated with regulating immune infiltration into the TME (24). Chen et al. (2019) created a novel *in vitro* p53 null murine gastric organoid model, containing epithelium with surrounding stroma, to study the gastric tissue microenvironment, in which M1-like macrophages, M2-like macrophages, and proliferating macrophages could all be identified by scRNAseq (52). This will serve as a valuable tool for understanding how distinct subsets of TAMs interact with the gastric epithelium. While these expected findings confirm that M1-like TAMs are associated with better outcomes than M2-like TAMs, the evolving scRNAseq technology holds promise to further delineate the different subsets of macrophages and their distinct roles in tumor progression in the coming years.

Aside from macrophage polarization, various groups have found specific mechanisms of how TAMs contribute to gastric carcinogenesis. TAMs were found to have enhanced gene expression profiles related to immunosuppression, including elevated levels of cytokines *IL-10* and *IL1B* (45). Another study found that epithelial-mesenchymal transition pathways were upregulated in TAMs in cancer tissue than the macrophages in adjacent tissue (22). When investigating various layers of tumor tissue, deep TAMs showed enrichment for *SPP1*. *SPP1*, also known as osteopontin, plays a role in regulating immune responses and has been found to be enriched in colorectal cancer TAMs, promoting tumorigenesis by interacting with fibroblasts and endothelial cells (71, 72). These observations give clues that TAMs may be driving gastric cancer progression by regulating proinflammatory immune cells and facilitating metastasis by enabling interactions between cancer cells and mesenchymal cells.

Dendritic cells (DC) are responsible for coordinating the immune response against tumor cells and have been identified in scRNAseq using *CD1C*, *CD83*, and *ADAMDEC1*. Although DCs are important immune cells, the current studies with scRNAseq data in gastric cancer immunity are yet to make a significant effort to understand the role of DCs. This is because DCs are not always identified within the datasets, and if they are, they are generally represented at a low frequency. Jiang et al. (2022) found the greatest population of DCs in metastatic lymph node derived tissue compared to primary tumor tissue (34). This is unsurprising since DCs typically reside in high frequency in the lymph node. These lymphoid derived DCs during metastatic disease were classified as plasmacytoid DCs and found to have primarily immunosuppressive functions. Jeong et al. (2021) found a DC cluster specific to tumor tissue but absent in normal adjacent tissue (21). Since the DC cluster lacked genes associated with classical and plasmacytoid DCs, they were thought to be new tumor-specific DCs. These tumor-specific DCs are enriched in deep tumor tissue, correlate with worse clinical outcomes of patients, and are thought to be activated by CCL2 within the TME to promote cancer progression. Likewise, Sathe et al. (2020) found a unique TME-associated DC population distinct from PBMC derived DCs, which highly expressed *IDO1*, a tryptophan catabolic enzyme associated with immunosuppressive cells (26, 73). DCs from cancer patient PBMCs were found to express more inhibitory receptors (*FTL*) and less proinflammatory chemokines (*CCL4, CCL5*) than healthy PBMCs (20). Contrary to many studies that assign DCs with a tumor-promoting role, Wang et al. (2021) found a myeloid DC fraction more amplified in less severe tumors than more severe ones (44). Taken together, these findings shed light on DCs as a mixed player in tumor immunity, i.e., depending on the phenotype and location, DCs may be either tumor-inhibiting or tumor-promoting. Future scRNAseq studies may need to isolate DCs from the TME to gain enough cells for clustering analysis, but this work, together with traditional techniques, will illuminate the intricate roles of DCs in gastric tumor progression.

Immature myeloid cells, such as myeloid derived suppressor cells (MDSC), have been identified in scRNAseq datasets using *S100a9*, *Ccr2*, *Cxcr2*. While MDSCs have been suggested to promote tumor growth and infiltration through suppressing the anti-tumor immune response, very few were identified in gastric cancer scRNAseq datasets (74). MDSCs were found in Nagaoka et al. (2020), Bockerstett et al. (2020), and Jiang et al. (2022) datasets but limited analysis was conducted to determine their specific role in gastric carcinogenesis (48, 54). Jiang et al. (2022) identified subtypes of MDSCs, phenotypically similar to either monocytes or granulocytes, which contributed to immunosuppression, tissue damage, and angiogenesis (34). Alshetaiwi et al. (2020) used scRNAseq to identify a specific immunosuppressive MDSC phenotype in models of breast cancer (75). These MDSCs could be distinguished by expression of *Agr2, Il1b*, and *Cd84*. Future work should establish whether this newly identified cancer localizing, immunosuppressive MDSC population plays a similar role in the setting of gastric cancer.

Neutrophils have previously been identified in the gastric cancer TME; however, granulocytes, such as neutrophils, are technically difficult to identify using current scRNAseq methods due to low RNA and high RNase content. Therefore, only a few scRNAseq studies looking in gastric cancer have discovered neutrophils (34, 44, 54). Nagaoka et al. (2020) identified neutrophils in scRNAseq data using *S100a8, Ccr1, and Cxcr2* and found that neutrophil recruitment into the TME promotes angiogenesis and epithelial-mesenchymal transition, suggesting neutrophils promote gastric tumor progression and metastasis (54). Similarly, Jiang et al. (2022) identified a *CXCR4* ^+^ neutrophil population enriched in metastatic tissue which was functionally expected to enhance tumor invasion (34). In this study, neutrophils expressing *PD-L1* were also found to directly interact with CTLs, promoting immune cell exhaustion. These findings are consistent with what has been shown in other cancer settings. Using scRNAseq in esophageal cancer, Yao et al. (2020) established that increased neutrophil proportion in the TME, *via* enhanced chemokine signaling, correlates with advancing cancer progression and found high levels of interaction between neutrophils and malignant epithelial cells (76). In gastric cancer, basal level epithelial cells produced high levels of *CXCL2* and *CXCL3*, which signal through *CXCR2*, likely recruiting and activating neutrophils during gastritis (29). In breast cancer, Szczerba et al. (2019) found that neutrophils escort circulating tumor cells (CTC) and promote cell cycle progression in CTCs, increasing the risk of metastasis (77). All of these findings together suggest that neutrophils promote progression and metastasis of many different cancers including gastric. In contrast, Wang et al. (2021) found an increase in neutrophils to be associated with the better prognostic, GI-mixed tumor lineage compared to the worse prognostic, Gastric-dominant tumor lineage (44). While this may contradict previous findings, it is not substantial enough to suggest an anti-tumor role for neutrophils in gastric cancer. Future improvement in single cell technology will hopefully allow for better transcriptional analysis of granulocytes to further determine the specific roles neutrophils play in promoting gastric cancer progression.

## Mast Cells and ILC2s

Seven groups independently identified mast cells in distinct scRNAseq datasets extracted from human gastric disease patients, using *TPSAB1* and *CPA3* (21, 24–26, 28, 29, 34). Yin et al. (2021) identified mast cells when pairing two previously published datasets together, and two other groups also identified mast cells within gastric diseased tissue from mice, using *Cpa3* and *Mcpt2* (39, 48, 50). This emphasizes that of the many datasets with mast cells present the majority are derived from human gastric cancer datasets. However, only a few studies analyzed them in detail in the setting of gastric disease. Yin et al. (2021) found a total of 370 mast cells after combining previously published datasets from various stages of the disease, from gastritis to cancer (39). Among these cells, they identified two unique mast cell clusters. Interestingly, one of these clusters had an increased presence in gastric cancer datasets with significant enrichment for *HDC* expression, which encodes histamine, a molecule that potentially promotes tumorigenesis (78). In Jeong et al. (2021), mast cells were more prevalent in normal adjacent and superficial gastric tumor tissues than in deeper gastric tumor tissues (21). These findings suggest that mast cells may surround the gastric tumors, promoting lateral tumor expansion into the neighboring healthy epithelial cells through histamine release. With such noteworthy observations from scRNAseq datasets, the lack of overall mast cell studies in this field warrants more in-depth research to better understand their role in promoting gastric cancer development.

ScRNAseq analysis also opened new avenues of research as some groups started focusing on the role of ILC2s (identified by *Gata3*, *Rora*, and *Il1rl1*) in mouse models of gastric tissue damage (50, 51). These studies stemmed from previous work that identified M2 macrophages as important instigators of the gastric carcinogenesis cascade in mouse models where the IL-33/IL-13 signaling network is required for initiation (61, 79). This led to the hypothesis that IL-13 is driving M2 recruitment/polarization in the gastric mucosa. IL-13 is thought to be produced by Th2 cells, but macrophages, mast cells, and ILC2s are also capable of producing IL-13, as observed in mouse models of gastric disease (49–51). Busada et al. (2019) used a mouse model where precancerous gastric lesions were induced by eliminating androgen and glucocorticoid signaling, inflicting gastric inflammation and tissue damage (50, 80). In this model, upon scRNAseq analysis of gastric infiltrating immune cells, ILC2s showed the highest levels of androgen and glucocorticoid receptors, suggesting that ILC2s stand to be severely impacted by a lack of these signalings (50). Further analysis of cytokine and cytokine receptor expressions in various immune cells found that ILC2s express *Il13* and *Csf2*, which may interact with IL-13 receptors found on macrophages, DCs, and fibroblasts or Csf receptors found on those same cells types with the addition of mast cells. The authors conclude that steroid hormones are important for suppressing inflammation mediated by ILC2s, which can promote gastric tissue damage *via* the recruitment and proliferation of tissue transformation driving immune cells. Meyer et al. (2020) isolated ILC2s from the stomachs of healthy and acutely drug-injured mice for scRNAseq (51). They found that ILC2s from damaged epithelium had increased expression of *Il4, Il5, Il13*, *Csf2, Pd1*, and *Ramp3*. They infer that gastric inflammation and tissue damage promote the recruitment of ILC2s into the gastric mucosa, which then produces cytokines capable of driving gastric carcinogenesis. Thus, these ILC2-focused studies believe ILC2s as the primary initiator of epithelial cell metaplastic transformation. Considering these new significant findings, it is imperative to establish the role of both ILC2s and mast cells in human gastric carcinogenesis and the mechanism of action of type two cytokines like IL-13. Future scRNAseq studies will need to directly compare different settings of gastric disease to determine if ILC2s and mast cells are common cell types promoting carcinogenesis or are setting specific.

## Fibroblasts and Endothelial Cells

Fibroblasts, identified by *DCN* and *COL4A1*, are known to facilitate cancer progression in many tumors. Their phenotype, location near the cancer tissue, and interactions with other cell types in the TME decide their impact on tumors. In general, gastric cancer-specific fibroblasts are more prevalent as the severity of the tumor is higher. They are also abundant in deep tumor tissues. These are termed cancer-associated fibroblasts (CAFs) and are known to interact with the cancer cells within tumor tissue constantly (21, 39, 44). CAFs show increased expression of genes that support tumor growth, such as genes involved in proliferation, angiogenesis, inflammation, and ECM remodeling compared to adjacent tissue fibroblasts (24). CAFs are also found to have elevated *ACTA2* and *EGR2*, promoting contractility and fibrosis within tumor tissue (26, 34, 35). CAFs specifically enriched in *INHBA*, a subunit of activin-inhibin complexes, and fibroblast activation protein (*FAP*) have a correlated increase with worsening tumor stage/grade and are associated with poor clinical prognosis (35). Thus, CAFs appear to have an overall pro-tumor role.

Two distinct fibroblast phenotypes were identified in scRNAseq gastric cancer datasets (23, 24, 34). One is an extracellular matrix cancer-associated fibroblast (eCAF), and another is an inflammatory cancer-associated fibroblast (iCAF). Fibroblasts matching the iCAF phenotype upregulate transcripts like *IL6, CXCL1*, and *CXCL12*. Enrichment of these genes and an identified proximity to exhausted CTLs in lymphoid structures suggest that iCAFs can regulate lymphocyte function (24). Kim et al. (2022) found that diffuse gastric cancer tissues showed a high prevalence of iCAFs compared to intestinal gastric cancer tissues, and overall iCAF number increased in gastric cancer compared to normal tissue (23). The same study also found a correlation between iCAFs and pro-stemness of tumors, suggesting that these cells promote cancer stem cell formation. Expression of *CXCL12*, a chemokine known to recruit lymphocytes, was also found to increase in fibroblasts with disease severity in a mouse model of esophageal cancer (76, 81). Qin et al. (2021) observed high *CXCL12* expressing fibroblasts within severe intestinal metaplasia tissue but not in early gastric cancer tissues; however, Jiang et al. (2022) found increased iCAFs in tumor tissue compared to normal adjacent tissue and that interactions between iCAFs and various immune cell subsets were mediated through *CXCL12-CXCR4* ligand-receptor pairing (34, 37). It is likely that CXCL12-producing iCAFs may play dual roles in recruiting immune cells and promoting their exhaustion. Yin et al. (2021) identified an iCAF-like population specific to chronic gastritis and intestinal metaplasia tissues that had upregulation of complement activation and apoptosis-associated genes (39). It is unclear whether iCAFs are more prevalent in precancerous lesions or continue to increase with cancer progression, but it can be said that iCAFs have the potential to regulate the immune response and promote an environment ideal for cancer development. The ECM-related CAFs, eCAFs, showed high expression of *POSTN*, a gene that supports epithelial cell adhesion and migration (24, 82). Distal tumor-adjacent tissue was more enriched for eCAFs over iCAFs, supporting the role for eCAFs enhancing the metastatic potential of gastric cancer. eCAFs were also shown to promote metastasis and even tumor growth through interacting with M2 macrophages *via* periostin (protein of *POSTN*). Of note, the expression level of *POSTN* has been associated with poorer survival outcomes (24). Kumar et al. (2022) and Jiang et al. (2022) both identified a CAF subset expressing muscle cell related transcripts (i.e., *ACTA2, TAGLN*) which likely represent a subset of eCAFs that promotes tumor invasion (34, 35). Further scRNAseq analysis and other works are needed to understand the roles of iCAFs, eCAFs, and other CAFs in gastric carcinogenesis. As evident from the development of specific organoid models, including stromal cells, efforts are underway to facilitate more studies on CAFs (35, 52).

Endothelial cells with markers *PECAM* and *VWF* are also identifiable in most scRNAseq studies. Tumor-derived endothelial cells (TDE) show unique gene expression profiles compared to tumor-adjacent endothelial cells (24). Studies show TDEs upregulate extracellular matrix genes and interact with CAFs *via VEGFA* receptor pairs, pointing to a pro-metastatic role (24, 26, 34). TDEs unique to gastric cancer and different from metaplasia show upregulated pathways associated with chemokines, metastasis, and cell cycle (39). Deep in diffuse tumors, inflammatory TDEs with increased expression of *IL6*, *ICAM1*, and *CCL2* are prevalent, suggesting a role for TDEs in immune regulation. Moreover, increased *CCL2* expression correlates with worse survival outcomes (21). Intestinal metaplasia-associated endothelial cells express *CXCL12* similar to iCAFs (37). Also, certain tumor-adjacent endothelial cells are significantly more enriched in genes associated with angiogenesis and cytokine production over TDEs (24). These findings suggest that peripheral or early tumor endothelial cells regulate immune cells to promote early tumor development. Li et al. (2022) found that endothelial to mesenchymal transitioning endothelial cells made a distinct cell subset in tumor tissue, suggesting TDEs not only promote tumorigenesis as a bystander, but also actively envelope the TME for enhanced growth (24). More research is needed to demystify how the various types of endothelial cells impact gastric cancer progression.

## Epithelial Cell Response to Inflammation

The cellular lineage of gastric adenocarcinoma is epithelial cells, specialized glandular cells lining the stomach wall. Therefore, it is critical to know how these cells respond to inflammation, which sets the course for gastric cancer development. In gastric tissue injury models, a precancerous lesion, referred to as spasmolytic polypeptide expressing metaplasia (SPEM), can be identified in two unique settings, *viz.*, acute drug injury and chronic autoimmunity, based on the differential expression of the SPEM-defining transcript spasmolytic polypeptide (also known as Trefoil Factor 2 or *TFF2*) (47). SPEM derived from both settings was found to be transcriptionally similar, but because the acute drug injury model has significantly less inflammatory infiltrate than the autoimmune model, the autoimmune-induced SPEM significantly upregulates several inflammatory transcripts (e.g., *Cd74, H2-Ab1*). Therefore, the autoimmune model is useful to understand how chronic inflammation triggers the transformation of healthy epithelial cells into inflamed precursor metaplastic cells that become SPEM (46). The findings from these studies suggest that inflammation actively contributes to epithelial cell transformation, initiating the pathological progression towards gastric cancer. Future scRNAseq work should compare SPEM arising out of other etiologies of gastritis, including the most common risk factor for gastric cancer, *Helicobacter pylori* infection (5). Noto et al. (2021) also found that IL-4 and/or IL-13, produced by mast cells or ILC2s, were required to drive the epithelial cell transition from precursor inflamed epithelial cells toward SPEM (49). Interestingly, in autoimmune animals that lack the IL-4/IL-13 receptors, making both epithelial and immune cells unable to respond to signaling, inflamed precursor epithelial cells arise but fail to progress to SPEM. Future studies may want to enhance IL-4/IL-13 signaling in models of autoimmune gastritis to determine how these inflammatory signals promote epithelial transformation.

IL-6/STAT3 signaling shows significant enhancement in tumor tissue in human gastric cancer datasets. STAT3 has been previously identified as an oncogene in gastric cancer from non-scRNAseq studies. STAT3 promotes gastric cancer cell proliferation, invasion, and chemoresistance (83). Through scRNAseq studies, we learn that IL-6, a cytokine that signals through STAT3, is enriched in malignant epithelium compared to non-malignant epithelium (28). Deep tumor tissue, where the immune response is exhausted and tumor growth is fostered, also has enhanced IL-6/STAT3 signaling, suggesting that this pathway favors carcinogenesis (21). Contrary to these data, Wang et al. (2021) found that, in long-term gastric adenocarcinoma survivors, this pathway was still enriched with other-immune-related pathways (i.e., IL-17 signaling, complement cascade, IFNα/γ signaling), suggesting a positive role in tumor immunity (44). Thus, the IL-6/STAT3 pathway appears to play a more complex role than just promoting cancer growth, even though several studies show a good correlation between activation of this pathway in epithelial cells and cancer progression. Transcriptomic studies focusing on STAT3/IL-6 signaling at various stages of gastric disease and cancer could improve mechanistic understanding of this pathway’s role in carcinogenesis.

Studies have found enhanced proinflammatory signatures in gastric cancer types with better prognoses. For example, EBV-derived gastric cancer clustered distinctively from worse prognosis gastric cancers with differential expression found in immune-related genes like Ly6 family members, antigen presentation molecules, and type I IFNs (28). GI-lineage tumors, which have a better prognosis than Gastric-lineage tumors, were found to be more immunologically active (44). It was also found that in diffuse gastric tumors there was a higher infiltration of immune cells, with enrichment for an exhaustive profile, than in the prognostically more favorable intestinal adenocarcinoma (24). Tong et al. (2022) discovered that tumors with a high autophagy signature score had dysfunctional T cell responses compared to low autophagy signature tumors which have a better prognosis; however, in contradiction to previous work, high autophagy score was also associated with low tumor expression of exhaustive transcripts (i.e., *CTLA4, PDL1*) (38). These combined findings suggest that gastric cancers with better survival outcomes overall have a proinflammatory antitumor phenotype and may be strong candidates for immunotherapy if possessing a low-moderate autophagy signature. Sundar et al. (2021) defined a unique profile of gastric tumors based on epigenetic changes in alternate promoter burden (APB) (31). APB^high^ tumors were found to have a poorer prognosis, depleted immune microenvironments, decreased immune checkpoint expression, and likely have enhanced immune evasion capabilities over APB^low^ tumors. This epigenetic profile was then found to be identified across a variety of cancer types suggesting that the ABP phenotype could be another strategy used to identify strong candidates for immunotherapy or a potential target for novel therapies. Some groups focused on identifying inflammatory signatures associated with worse prognostic tumors. In an organoid model, diffuse gastric cancer showed enrichment for chemokines, such as *CXCL3*, *CXCL5*, *CXCL7*, which have been associated with malignant progression (53, 84). In metastatic tumors, transcription factors *FOS* and *JUN* were associated with metastatic progression and kinases *ERBB2* and *CDK12* could identify metastatic tissue compared to primary tumor tissue (27). Wang et al. (2021) found that tumor cell surface expression of the molecules CMTM4/CMTM6 positively regulates expression of PD-L1 (42). Co-expression of CMTM4/6 with PD-L1 correlated with poorer prognoses, but these patients have higher early efficacy with immune checkpoint blockade therapy, making these molecules a potential prognostic indicator for favorable treatment. Zhao et al. (2021) also identified four novel diagnostic and prognostic gastric cancer biomarkers using scRNAseq including *BGN*, *COMP*, *COL5A2*, and *SPARC* (43). Enriched expression of these transcripts was associated with increased immune cell infiltration and drug sensitivity as well as decreased survival outcomes. Taken together, epithelial cells seem to mount multifaceted responses to inflammation, and further explorations across adenocarcinoma settings will likely unravel the complexities, identifying novel diagnostic and therapeutic targets for improving clinical outcomes.

## Tools for Analyzing Gastric Cancer ScRNAseq Data

**Figure 2:**
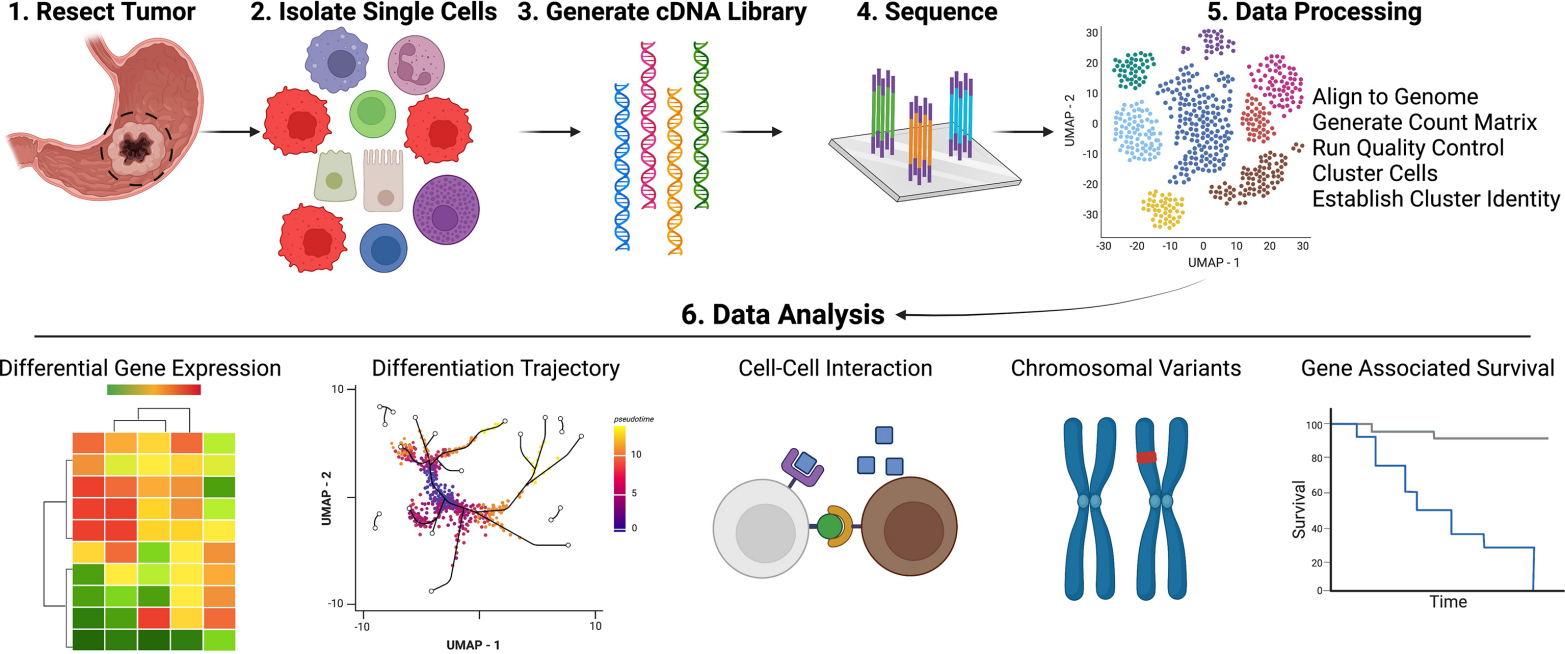
Pipeline for Acquisition and Analysis of Single Cell Data. Created with Biorender.com.

Several reviews have provided overviews of currently available scRNAseq technologies with exhaustive lists of available analytical tools. Therefore, in this review, we focus on the scRNAseq tools relevant to gastric cancer datasets (12, 13, 15, 85). An overview illustrating the general pipeline, workflow for scRNAseq can be found in **Figure 2**. To run scRNAseq on any tissue, single cells must first be disassociated from the tissue and then thoroughly filtered to avoid clogs and to assure isolation of transcripts from single cells. For gastric tissue processing, either human or mouse, tissue must undergo two rounds of digestion to disassociate glands and then single cells. Organoids require only a single digestion step to disassociate glands (53). Groups that utilize liquid biopsies from metastatic ascites fluid can avoid this double digestion step and simply run centrifugation and clean-up for single cell isolation (44, 45). Isolated single cells, which may be sorted into specific populations (i.e., immune cells or epithelial cells) for enriched datasets, are then ready to undergo scRNAseq library generation. Among the several technologies available, the most common one is the microfluidic, droplet-based method available through 10X Genomics (Pleasanton, CA) (86). 10X Genomics also offers a VDJ kit for amplifying TCR or BCR sequences within scRNAseq datasets for downstream clonotype analyses. For TCR and BCR data visualization, Jia et al. (2021) plotted the number of unique clonotypes, overall clonotype abundance, and clonotype frequency within various cell subsets as well as utilized Circos plots to annotate receptor sharing across T and B cell subsets (22, 87). Other prominent library generation techniques utilized for gastric cancer datasets include FACs-based isolation *via* CEL-seq2, SMART-Seq2, and BD Rhapsody, as well as a targeted gene expression vertical flow array chip (VFAC) system (54, 88–91). Several current reviews compare the different scRNAseq methodologies, providing further insight for experimental design and approach (12, 14, 15, 18, 85, 92). After libraries are constructed, the sequenced data can be extracted for QC and analysis. 10X Genomics provides their own software, CellRanger, which works seamlessly with their library generation methods. With CellRanger, raw sequencing data can be aligned and converted into count matrix files, which are then used in downstream analyses. Methods for FACs-based single cell prep are less simplified, but programs like STAR can be used to align transcripts to a genome of interest, htseq-count can be utilized to generate a count matrix, and scran and/or scater conduct normalization of mapped reads (27, 56, 58, 93–96). For those more familiar with R language, Seurat, created by the Satija lab, can be utilized for QC, integration, dimensionality reduction, clustering, and differential gene expression analysis (97, 98). For those more comfortable with the python language, SCANPY is another software available for preprocessing, clustering, and differential gene expression analysis of scRNAseq data (99).

After generating unbiased cell clusters within scRNAseq datasets, analyzing and assigning cell identity to the established cluster profiles is an essential step. A summary of tools and programs useful to analyze gastric disease datasets is presented in **Table 1**. As evident from this Table, various groups have used a variety of programs specific to their needs to assign or estimate cell identity. Aran et al. (2019) developed SingleR program that automatically annotates scRNAseq data (21, 22, 100). It labels the identity of new cells from a test dataset based on similarity to the reference, primarily obtained from bulk RNAseq data. DynamicTreeCut is a dynamic tree-cutting approach for assigning cell types to a dendrogram of hierarchical clustering (58, 100, 101). Of note, CIBERSORT is a valuable tool that has helped researchers to make use of scRNAseq analyses to expand bulk RNAseq findings, utilizing scRNAseq transcriptomic profiles to infer cell-type composition that is lost in the bulk datasets (38, 44, 103). Wang R, et al. (2021) used the human cell lineage (HCL) database through the package scHCL to estimate the origin epithelial cell types of different gastric cancer clusters (44, 102). Gene Ontology (GO) analysis is a widely used effective tool for estimating the cellular function of identified clusters based on gene expression. The molecular signature database (MSigDB), accessible through the Gene Set Enrichment Analysis (GSEA) website, offers a vast number of curated gene sets available for download to conduct pathway analysis (106). Some of the databases utilized for pathway analysis in gastric cancer research include Hallmark, REACTOME, Kyoto Encyclopedia of Genes and Genomes (KEGG), Aung et al. (2006) gastric cancer enriched gene library and the recently developed Human Autophagy Database (HADb) (38, 111, 112, 135). Multiple gene annotation programs are currently being used to conduct GO analysis, *viz.*, EnrichR, Metascape, clusterProfiler, WebGestalt, and GSVA (104, 105, 107–110). Other cell enrichment programs specific for identifying cell state, regulatory gene networks, and cell cycle score are SCENIC and CellCycleScoring (found in the Seurat Package) (24, 26, 39, 44, 56, 59, 97, 113). To infer intercellular interactions and communications between clusters, the following programs are applied: FANTOM5, STRING, CellPhoneDB, and CellChat (114–118). Many groups choose to conduct cellular differentiation “pseudotime” trajectory analysis using various programs, such as Monocle2 and 3, Slingshot, SCORPIUS, and MarkovHC (119–122). RNA velocity is another trajectory program that can estimate cellular differentiation state based on percentages of unspliced to spliced mRNAs (123). Of important consideration for scRNAseq datasets, the spatial context within tissue is lost due to the nature of sample preparation. To address this, Jia L, et al. (2021) used CSOmap to computationally reconstruct cellular spatial organization within the tissue (22, 136). Jiang et al. (2022) also utilized NanoString’s GeoMx Digital Spatial Profiler to expand upon scRNAseq results, identifying the spatial relationships and further gene expression differences between previously identified cell subsets (35). We expect that the future gastric cancer-focused works will likely incorporate this kind of direct rather than inferred spatial information with scRNAseq.

**Table 1:** Summary of analytical tools available for gastric cancer scRNAseq data.

Analysis Type	Program	Datasets
**Cell Identity**	SingleR (100) dynamicTreeCut (101) scHCL (102) CIBERSORT (bulk RNA seq) (103)	HCL (102)
**Gene Ontology**	EnrichR (104, 105) Metascape (107) clusterProfiler (108) GSVA (109) WebGestalt (110)	MSigDB (106) Hallmark (111) REACTOME (112) KEGG HAdb (38)
**Cell State**	SCENIC/ (113) CellCycleScoring (97)	
**Intercellular Interaction**	FANTOM5 (114) STRING (115) CellPhoneDB (116, 117) CellChat (118)	
**Trajectory**	Monocle2/3 (119) Slingshot (120) SCORPIUS (121) Markov HC (122) RNA velocity (123)	
**Chromosomal Variation**	inferCNV (44, 124) LIAYSON (55) VarTrix (23) GISTIC2 (125) Mutect2 (126) CopyKAT (127)	
**Survival**	Survminer (29) KaplanMeier Plotter (128)	
**Cancer Gene Expression**	CIPHER (129) CancerSEA (130) GEPIA2 (131) UCSC Xena (132)	TCGA STAD (133) Firebrowse (134) PanCanAtlas

Programs and datasets listed are cited with either original publications or a publication that utilized this technology for analysis. Links to datasets can be found in **Supplementary Table 1**.

Groups working with human gastric cancer datasets can utilize multiple analysis programs to estimate cancer severity and associated high-risk expression patterns. Various groups attempted to analyze chromosomal alteration, such as copy number variants (CNV) present within tumor tissue, to find relevant and reproducible mutations associated with gastric cancer. CNVs can be estimated by averaging relative gene expression levels over their respective genomic regions. The program inferCNV, available through the Broad Institute, has been used by several groups to identify copy number alterations of malignant cells within scRNAseq data (18, 21, 22, 44, 45, 124, 137). Many other programs are also useful to estimate chromosomal alterations within cancer scRNAseq datasets, *viz.*, LIAYSON, 10X supported VarTrix, GISTIC2, CopyKAT, and Mutect2 (23, 25, 44, 55, 125–127). ABSOLUTE can be used to infer intratumoral heterogeneity (28, 128, 138). As well, the TIMER tool is a comprehensive resource designed to analyze immune cell infiltrates across diverse cancer subtypes (139). The TCGA has a publicly available human stomach adenocarcinoma (STAD) dataset containing clinical annotations along with molecular profiles that were utilized as a reference or primary dataset in several studies (25, 28, 36, 37, 41, 45, 133). FirebrowseR, also generated by the Broad Institute, is a database which provides access to the PanCanAtlas, containing conserved cancer gene expression data (134). Kaplan-Meier survival curves for gastric cancer datasets were generated based on differential gene expression using programs like the survival and survminer R packages and Kaplan-Meier Plotter. CellMiner is another valuable database and web tool designed to facilitate estimations in pharmacological sensitivities of various cancer cells (140). Other programs are also available for determining differential gene expression in high-risk gastric tumors or other cancers, including CIPHER, CancerSEA, UCSC Xena and GEPIA2 (129–132).

Some of the above tools were adapted from bulk RNA sequencing (e.g., EnrichR, FANTOM5, MSigDB), while others were specifically designed to address the challenges of single-cell analysis (e.g., SCENIC, CellPhoneDB, inferCNV). ScRNA technology in gastric cancer is advancing rapidly, and by combining different programs, scRNAseq data can now be applied to cancer cell profiling and risk assessment strategies. The future of scRNAseq technology in gastric cancer appears promising for developing precision medicine.

## Discussion

ScRNAseq technology focused on gastric cancer and immune-mediated mechanisms of carcinogenesis have begun to advance the field greatly in a short period. We have learned that T cells may be important for initiating tissue damage, but after cancer arises, exhaustion limits their protective role (21, 24, 38, 48, 54). New findings that NKT cells as a possible long-lasting cell type that promotes antitumor immunity will undoubtedly drive future studies (22, 25). B cells appear to be prominent in the early stages of the disease, possibly promoting cancer development in an autoimmune setting or limiting cancer progression through complement activation (9, 22, 48, 64). However, as cancer progresses, the TME induces B cell apoptosis, limiting their role in advanced gastric cancer (22, 24, 39). Like in many cancers, scRNAseq results reveal M1 macrophages as prominent antitumor immune cells while M2 macrophages support gastric tumor growth (44, 45, 52). Unlike in other forms of analysis, M1 and M2 phenotypes are not clearly identifiable with scRNAseq. In fact, studies reveal that there could be multiple distinct subsets of macrophages playing opposing roles in tumor immunity, an interesting finding that may change our view in other cancers as well (21, 26, 39, 45). Dendritic cells and immature myeloid suppressor cells are not prevalent in these gastric cancer datasets. Hence, their role in gastric cancer is still obscure, warranting a need for more advanced research in the future (19, 20, 25, 54). Neutrophils were also not commonly identified, as granulocytes do not always make it through scRNAseq processing, but likely play a protumor role in disease (54). In contrast, mast cells were identified in multiple datasets, but still, there are many unanswered questions regarding their role in tumor development (21, 24–26, 28, 29, 39, 48, 50). Interestingly, ILC2s have been implicated as being responsible for driving early tissue damage and recruiting destructive inflammatory cells; however, more studies in human gastric cancer datasets are needed (50, 51). Both ILC2s and mast cells can produce the cytokine IL-13, which has been associated with driving epithelial metaplastic transformation (49). Further work is required to establish the roles of both of these cell types early on in cancer development. Other stromal cells within the TME, like fibroblasts and endothelial cells, also likely play immunological roles in tumor progression. CAFs and endothelial cells have been found to play distinct roles in regulating the antitumor immune response and promoting cancer growth and metastasis (23, 24, 26, 39). Epithelial cell responses to these various inflammatory signals have displayed mixed findings with regards to promotion and inhibition of tumor progression, especially when focusing on different time points of disease and distinct gastric cancer types. Further work on understanding the epithelial cell response to inflammation is needed to determine the mechanisms of cancer development and progression. In summary, a wealth of new information has been obtained using scRNAseq technology including a better understanding of the immunological landscape and roles of distinct immune cells within the gastric cancer TME. Although we are still in the early stages of using this technology in gastric cancer, it has already stoked poignant questions and opened new avenues of research. The current trend clearly points to the robust growth of this technology in this field and promises to shed more light on the immunological mechanisms underlying gastric carcinogenesis.

In the future, scRNAseq can be utilized to significantly advance the field of gastric cancer. Specifically, scRNAseq can be utilized on immune cells surrounding and within gastric tumor samples to identify the inflammatory infiltrate profile at different stages of disease. This will help determine the type of inflammation associated with increased risk of cancer progression. Novel therapeutics can be identified through scRNAseq, finding new immune cells for immunotherapy targets. As well, scRNAseq can help transcriptionally define precancerous lesions with higher risk of progressing to cancer. Among the datasets selected for this review, there was little inclusion of spatial information, but future work will likely harness spatial transcriptomics for single-cell analysis (141). Incorporating spatial transcriptomics with single-cell technology will allow for an enhanced understanding of intertumoral interactions and structure. New frontiers in single cell transcriptomics combine tissue morphology (spatial transcriptomics), cell-surface protein expression (CITE-seq), and CRISPR screening technology (CROP-seq) with scRNAseq to improve and increase the methodologies for revealing immune mediated mechanisms of gastric carcinogenesis (142–146). Expanding beyond the field, this technology can be used to identify shared features across tumor environments from different cancer settings to try and discover pan-cancer phenotypes/targets/inflammatory profiles.

While the scRNAseq technology has proven incisive and powerful in providing new insights into gastric cancer initiation and progression, there are many challenges to applying this technology. Challenges with scRNAseq include a low sequencing depth achieved when compared to bulk sequencing depth, relatively high cost, low sample numbers, lack of detection for lower abundance transcripts, need for fresh tissue samples, certain cell type bias (granulocytes are typically difficult to target due to low RNA and high RNase content) and potential differences in expression profiles when capturing transcripts from either the 3’ or 5’ end with a predominance in datasets generated using the 3’-targeted technology. These challenges add to the ongoing debate of whether scRNAseq findings require validation by alternative wet-lab approaches (e.g., qPCR, immunostaining, RNA *in situ* hybridization) which can increase costs and time to publication. How often and in what contexts scRNAseq findings need confirmation remains unstandardized, but when emphasizing a select few genes it is advisable to validate with alternative methods (147). Furthermore, the rapid pace at which this field is growing makes it difficult for many researchers (more so clinicians) to keep up with the advances. While all these issues may look daunting, the overall need for this technology and its capability to advance the field is also overwhelming. Never before has it been possible to unbiasedly identify transcriptional changes at a single-cell resolution and gain vast amounts of information from small samples. Once costs are reduced, workflows standardized, and accessibility to clinical research is streamlined, scRNAseq can be effectively utilized for early detection, diagnosis, risk stratification of cancer patients, accelerated drug discovery, and gastric cancer prevention.

## Author Contributions

SH substantially contributed to the concept and outline of the work. SH also drafted and revised the work for publication. RD revised, read, and approved the manuscript for submission. MP read and revised drafts of the work. All authors contributed to the article and approved the submitted version.

## Funding

This work was funded by the National Institutes of Health, National Institute of Diabetes and Digestive and Kidney Diseases (NIDDK), an R56 (2R56DK110406-06A1) and RO1 (RO1DE025141).

## Conflict of Interest

The authors declare that the research was conducted in the absence of any commercial or financial relationships that could be construed as a potential conflict of interest.

## Publisher’s Note

All claims expressed in this article are solely those of the authors and do not necessarily represent those of their affiliated organizations, or those of the publisher, the editors and the reviewers. Any product that may be evaluated in this article, or claim that may be made by its manufacturer, is not guaranteed or endorsed by the publisher.
