# Supplementary material for: Discovering Immune-Mediated Mechanisms of Gastric Carcinogenesis Through Single-Cell RNA Sequencing
Source: Front Immunol. 2022 Jun 10;13:902017. doi: 10.3389/fimmu.2022.902017 (PMC9231461; doi:10.3389/fimmu.2022.902017)
Supplement: Supplementary file 1 [file Table_1.pdf]

Analysis Type	Program	Datasets
**Cell Identity**	SingleR (Aran D, et al. 2019)<br>dynamicTreeCut (Langerfelder P, et al. 2008)<br>scHCL (Han X, et al. 2020)<br>CIBERSORT (bulk RNA seq) (Newman AM, et al. 2015)	HCL (Han X, et al. 2020)
**Gene Ontology**	EnrichR (Chen EY, et al. 2013, Kuleshov MV, et al. 2016)<br>Metascape (Zhou Y, et al. 2019)<br>clusterProfiler (Yu G, et al. 2012)<br>GSVA (Hänzelmann S, et al. 2013)<br>WebGestalt (Wang J, et al. 2017)	MSigDB (Subramanian A, et al. 2005)<br>Hallmark (Liberzon A, et al. 2015)<br>REACTOME (Croft D, et al. 2014)<br>KEGG<br>HAdb (Tong T, et al. 2021)
**Cell State**	SCENIC (Aibar S, et al. 2017)<br>CellCycleScoring (Butler A, et al. 2018)	
**Intercellular Interaction**	FANTOM5 (Ramilowski JA, et al. 2015)<br>STRING (Szklarczyk D, et al. 2019)<br>CellPhoneDB (Vento-Tormo R, et al. 2018, Elfremova M, et al. 2020)<br>CellChat (Jin S, et al. 2021)	
**Trajectory**	Monocle2/3 (Trapnell C, et al. 2014)<br>Slingshot (Street K, et al. 2018)<br>SCORPIUS (Cannoodt R, et al. 2016)<br>MarkovHC (Wang Z, et al. 2022)<br>RNA velocity (La Manno, et al. 2018)	
**Chromosomal Variation**	inferCNV (Patel AP, et al. 2014, Wang R, et al. 2021)<br>LIAYSON (Andor N, et al. 2020)<br>VarTrix (Kim J, et al. 2022)<br>GISTIC2 (Mermel CH, et al. 2011)<br>Mutect2 (Cibulskis K, et al. 2013)<br>CopyKAT (Gao R, et al. 2021)	
**Survival**	survminer (Zhang P, et al. 2019)<br>KaplanMeier Plotter (Győrffy B, et al. 2010)	
**Cancer Gene Expression**	CIPHER (Wu X, et al. 2008)<br>CancerSEA (Yuan H, et al. 2019)<br>GEPIA2 (Tang Z, et al. 2019)<br>UCSC Xena (Goldman M et al. 2020)	TCGA STAD (Liu J, et al. 2018)<br>Firebrowse (Deng M, et al. 2017)<br>PanCanAtlas
